# Highly Effective Modulator Therapy in Cystic Fibrosis: Addressing Unusual Variants in the Middle East

**DOI:** 10.1155/pm/3622052

**Published:** 2025-12-26

**Authors:** Said Isse, Ali Saeed Wahla, Mateen Haider Uzbeck, Zaid Zoumot, Mohamed Abuzakouk, Irfan Shafiq

**Affiliations:** ^1^ Respiratory Institute, Cleveland Clinic Abu Dhabi, Abu Dhabi, UAE, clevelandclinicabudhabi.ae; ^2^ Department of Allergy & Immunology, Cleveland Clinic Abu Dhabi, Abu Dhabi, UAE, clevelandclinicabudhabi.ae

## Abstract

**Background:**

Cystic fibrosis (CF) is an autosomal recessive disorder caused by variants in the CFTR gene. Although the F508del mutation is common globally, the Middle East exhibits a higher prevalence of rare, region‐specific variants. The triple‐combination therapy elexacaftor/tezacaftor/ivacaftor (ETI) has revolutionized CF management; however, its efficacy in individuals with rare variants, often underrepresented in clinical trials, remains less certain. This study is aimed at evaluating the real‐world outcomes of ETI therapy in CF patients with rare CFTR variants predominantly found in the Middle East.

**Methods:**

This retrospective, single‐center study included 12 patients with CF carrying rare Middle Eastern variants. Data on percent predicted Forced Expiratory Volume in 1 second (ppFEV1), body mass index (BMI), and annual exacerbation frequency were collected before and after 12 months of ETI treatment. Nine of these patients were previously on ivacaftor and were switched to ETI. Changes in clinical outcomes were analyzed using Wilcoxon signed‐rank tests due to nonnormally distributed data.

**Results:**

Following 12 months of ETI therapy, significant improvements were observed. The median ppFEV1 increased by 9.5% (range: 2–15). The median annual frequency of exacerbations decreased by two events (range: 0–4). BMI showed a modest median improvement of 1.5 kg/m^2^, which was not statistically significant. The cohort comprised nine females (75%) and three males (25%), with a median age of 24.3 years (range: 18.5–35.2 years) at the time of ETI initiation or transition.

**Conclusion:**

ETI therapy led to statistically significant improvements in lung function and a reduction in pulmonary exacerbations in CF patients with rare Middle Eastern variants. These findings, from the first report of its kind in this region, support the expansion of ETI access to individuals with rare CFTR variants, particularly in underserved populations, based on functional response. This underscores the benefit of ETI beyond the common F508del mutation.

## 1. Introduction

Cystic fibrosis (CF) is a rare, autosomal recessive disorder caused by variants in the CF transmembrane conductance regulator (CFTR) gene, resulting in defective ion transport across epithelial membranes [1]. CF is characterized by thick, sticky secretions produced by the glands, leading to a multisystem disease that most severely affects the respiratory, digestive, and reproductive systems. Progressive respiratory failure remains the most common cause of death [2]. Significant other manifestations leading to morbidity in CF patients include exocrine pancreatic insufficiency, malnutrition, and diabetes [1].

The F508del remains the most common CF mutation worldwide, accounting for approximately 80% of all CF alleles [3]. However, our previous research has shown CF mutation patterns observed in the adult Middle Eastern cohort differ significantly from published data typically found in Western populations. The UAE population demonstrated a low F508del prevalence. Instead, the S549R mutation was the commonest, found in 28% of patients (11 out of 39), followed by ΔF508 (23% of patients, *n* = 9) and the 3849+10 kbC > T mutation (*n* = 9). This pattern highlights the presence of rare variants more specific to this region. Additionally, due to the high level of consanguinity in the UAE, the cohort showed a high rate of homozygosity for CF variants, with only 6 out of 39 patients being heterozygotes [4].

Survival in CF has improved significantly by implementing optimized treatments—such as long‐term antibiotics, mucolytics, nutritional support, and bronchopulmonary hygiene—delivered through multidisciplinary teams in specialized care centers [5]. However, the advent of CFTR modulators, particularly the triple‐combination therapy elexacaftor/tezacaftor/ivacaftor (ETI), has dramatically changed the prognosis and management of CF by directly targeting CFTR′s protein defects. Although ETI has shown considerable efficacy in patients with at least one F508del allele and many other Class II–IV variants [5, 6], its benefit for individuals who carry rare or unclassified variants, often underrepresented in clinical trials, is still uncertain.

Here, we present real‐world outcomes of ETI in a case series of individuals with rare CFTR variants predominantly found in the Middle East. The study evaluates pretreatment and posttreatment changes in lung function, nutritional status, and exacerbation frequency, highlighting the importance of extending precision medicine approaches to patients with rare variants.

## 2. Methods

This retrospective, single‐center study was conducted at the Cystic Fibrosis Clinic, Cleveland Clinic Abu Dhabi. All consecutive people with CF (pwCF) with rare CFTR variants who were initiated on ETI between January 2022 and December 2023 were included. No eligible pwCF were excluded.

Ethical approval for the study was obtained from the Cleveland Clinic Abu Dhabi Institutional Review Board. All data were anonymized prior to analysis.

Demographic and clinical data were extracted from electronic medical records, including age, sex, genotype, and prior CFTR modulator therapy. For each participant, body mass index (BMI), percent predicted Forced Expiratory Volume in 1 second (ppFEV1), and the annualized frequency of pulmonary exacerbations were recorded for 12 months before and 12 months after the initiation of ETI.

Exacerbations were defined as episodes requiring oral or intravenous antibiotics for worsening respiratory symptoms.

ETI was prescribed either as a step‐up from ivacaftor for gating variants or initiated off‐label in pwCF with rare or minimal‐function variants demonstrating clinical decline.

The data were analyzed using SPSS Version 27. Descriptive statistics were calculated for baseline and posttreatment measurements of BMI, ppFEV1, and annual exacerbation frequency, including means, standard deviations, and minimum and maximum values. Differences between pretreatment and posttreatment values were computed for each outcome variable. The normality of these differences was assessed using Shapiro–Wilk test. Due to nonnormal distributions in some variables, nonparametric tests were employed for further analysis. Changes in outcomes following ETI treatment were evaluated using Wilcoxon signed‐rank tests chosen for their suitability in analyzing paired, nonnormally distributed data. Statistical significance was defined as a two‐tailed *p* value of less than 0.05.

## 3. Results

**Figure 1:**
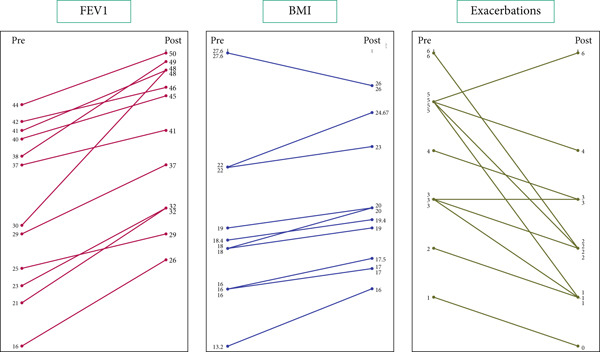
Slopegraph showing individual outcomes pretreatment/posttreatment.

The study included 12 pwCF with rare Middle Eastern CFTR variants (Table 1). The cohort comprised nine females (75%) and three males (25%), with a median age of 24.3 years (range 18.5–35.2 years) at ETI initiation. The variants present in study participants are listed in Table 2, whereas the outcomes for individual patients are charted in Figure 1 and Table S1. Of the 12, nine had previously received ivacaftor and transitioned directly to ETI. The remaining three were modulator‐naïve. Table S1 has all individual observations for each patient.

**Table 1:** List of mutations in the case series.

**Patients**	**Gender**	**Mutation 1**	**Mutation 2**
1	F	1548delG	1548delG
2	F	3120+1G>A	3120+1G>A
3	M	3120+1G‐>A	3120+1G‐>A
4	F	406‐2A>G	406‐2A>G
5	F	G27V	G27V
6	F	3849+10kbC>T	R1158X
7	M	S549R	S549R
8	M	S549R	S549R
9	F	S549R	S549R
10	F	S549R	S549R
11	F	S549R	S549R
12	F	I1234V	I1234V

**Table 2:** Mutation classes in the case series.

**Mutation**	**Class**	**Effect**
406‐2A>G	I	Protein production defect (nonsense mutation, no protein)
3120+1G>A	I	Protein production defect (minimal function/nonsense mutation, no protein)
1548delG	I	Protein production defect (frameshift mutation, no protein)
R1158X	I	Protein production defect (nonsense mutation, no protein)
S549R	III	Gating defect (protein at surface but defective opening)
I1234V	IV	Gating defect (protein at surface but defective opening)
2789+5G>A	V	Insufficient protein (splicing, reduced protein)
3849+10kbC>T	V	Insufficient protein (splicing, reduced protein)
G27V	Unclassed mutation	

After 12 months of ETI, mean ppFEV1 increased by 9.5% (range: 2%–15%). The annual frequency of exacerbations decreased by a mean of two events (range: 0–4), and BMI increased by a mean of 1.5 kg/m^2^, which was modest but clinically meaningful.

Among pwCF previously on ivacaftor, mean ppFEV₁ rose from 33% to 42% predicted, and BMI increased by 1.3 kg/m^2^ (added). In contrast, modulator‐naïve individuals achieved a mean ppFEV1 improvement from 29% to 39%, indicating substantial functional gains even without prior modulator exposure.

Normality testing revealed that ppFEV1 differences approximated a normal distribution (Shapiro–Wilk *p* = 0.081), whereas BMI changes deviated (*p* = 0.018), supporting nonparametric testing. The Wilcoxon signed‐rank test confirmed statistical significance for ppFEV1 (*Z* = –3.065, *p* = 0.002) and for exacerbation reduction (*Z* = –2.675, *p* = 0.007).

Subgroup analysis demonstrated that the Class I variants (*n* = 4) and non‐Class I variants (*n* = 8, including gating and residual‐function alleles) both exhibited clinically relevant improvements in ppFEV1, BMI, and exacerbation rates (added). Individual‐level outcomes for each pwCF, including genotype, prior modulator status, and pre–post treatment changes in ppFEV1, BMI, and exacerbations, are presented in Table 3.

**Table 3:** Summary comparison table.

**Variable**	**ETI**	**All patients**	**Class 1**	**Nonclass 1**	**Switching from ivacaftor**	**Ivacaftor naïve**
*n*		12	4	8	9	
BMI (kg/m^2^)	Pre	13.2–27.6, 19.5 (4.5)	16–23, 18.8 (3.1)	13.2–26, 19.2 (4.5)	13.2–27.6, 19.2 (4.6)	16–27, 19.3 (4.8)
	Post	16.0–26.0, 20.5 (3.6)	17–26, 21.0 (3.7)	16–27.6, 20.5 (3.7)	16–26, 20.5 (3.7)	17–26, 19.9 (3.6)
FEV1 (% predicted)	Pre	16–44, 32.2 (9.4)	16–41, 28.8 (12.1)	23–44, 33.9 (8.0)	23–44, 33.0 (8.1)	16–41, 29.0 (10.5)
	Post	26–50, 40.3 (8.7)	26–48, 36.9 (9.7)	29–50, 42.0 (8.2)	29–50, 42.0 (8.1)	26–48, 39.0 (9.8)
Exacerbations (events/year)	Pre	1–6, 4.0 (1.6)	5–6, 5.3 (0.5)	1–6, 3.4 (1.6)	1–6, 3.4 (1.6)	4–6, 5.0 (0.7)
	Post	0–6, 2.3 (1.6)	1–6, 3.3 (2.2)	0–3, 1.8 (1.1)	0–3, 1.8 (1.0)	1–6, 3.2 (1.9)

*Note:* Values are presented as minimum–maximum, mean (SD). Exacerbations are reported as annualized number of events. Pre refers to measurements prior to initiation of treatment; Post refers to measurements after treatment.

Abbreviations: BMI, body mass index; FEV1, forced expiratory volume in 1 second (percent predicted).

## 4. Discussion

This retrospective, single‐center, real‐world study demonstrates that initiation or switching to ETI led to significant improvements in lung function and reduced pulmonary exacerbations among pwCF with rare CFTR variants predominantly seen in the Middle East. To our knowledge, this is the first report from the region providing real‐world outcome data in pwCF with uncommon or unclassified CFTR variants.

Our findings underscore the feasibility and clinical benefit of modulator therapy even in genotypes not yet fully characterized by pivotal clinical trials. The ppFEV1 increased by a median of 9.5% in our cohort; while there is no widely accepted definition of minimal clinically significant difference in FEV1 value in CF, a commonly accepted threshold is an increase of 5% or more from baseline. Previously, Nichols et al. showed in the PROMISE study, a postapproval observational study that included a diverse population, an average ppFEV1 improvement of 9.8% from baseline at 6 months into ETI therapy in individuals aged 12 years and older [7].

Although the improvement in BMI did not reach statistical significance, the absolute increase from 19.4 kg/m^2^ to 20.5 kg/m^2^ in our cohort was similar to the findings from the study by Burgel et al., who reported a mean BMI increase from 19.9 kg/m^2^ to 20.8 kg/m^2^ at 3 months in patients with at least one rare, non‐F508del CFTR mutation. This highlights the nutritional benefits of ETI even in diverse populations [8].

The frequency of annual exacerbations decreased by two (0–4) events in our cohort; this is similar to a retrospective study involving 16 CF patients with non‐F508del variants, which showed the median annual rate of pulmonary exacerbations decreased from 1.5 to 0 after 3–6 months of ETI treatment [9].

Importantly, subgroup analysis revealed that both ivacaftor‐experienced and modulator‐naïve pwCF benefitted from ETI, indicating a true pharmacologic effect rather than a residual benefit from prior therapy. These findings support the hypothesis that ETI may enhance CFTR function even in certain splice or nonsense variants with minimal baseline activity.

Overall, our findings align with a recent systematic review evaluating the real‐world clinical effectiveness of using ETI in non‐F508 variants. This review showed that 37 of the 38 individuals who were FDA‐approved non‐F508 variants showed clinical responses to ETI [10]. Likewise, our results are consistent with real‐world registry data from Germany, which reported similar improvements in FEV1, BMI, and exacerbations following ETI initiation [11].

Our study includes a range of rare variants, some unique to this region. These variants range across the spectrum of different classes, with previous research from the UAE showing a higher burden of rare splicing and nonsense variants [4].

ETI has shown limited efficacy in individuals with CF carrying Class I CFTR variants, typically resulting in little to no functional CFTR protein due to premature stop codons or splicing defects. However, emerging research indicates that certain minimal function or splicing variants may exhibit some responsiveness to ETI; in a study using patient‐derived intestinal organoids (PDIOs) Kreos et al. suggest that individuals harboring genotypes that include these variants may exhibit responsiveness to ETI [12]. These findings complement our clinical results by suggesting that even minimal‐function or splicing variants may exhibit partial modulator responsiveness when exposed to ETI.

Furthermore, recent studies support the functional efficacy of ETI in patients with non‐ F508del variants previously considered minimally responsive. A 2024 United States multicenter trial demonstrated significant clinical improvement in individuals carrying the N1303K mutation [13]. Another study by Fainardi et al. confirmed the clinical benefits of ETI in pediatric and adolescent patients with rare variants not currently included in FDA‐approved labels, supporting off‐label consideration in functionally characterized responsive genotypes [14].

Nevertheless, recent real‐world data from the French compassionate program, where 95% of pwCF with two “true” Class I CFTR variants showed no clinically meaningful response to ETI and were classified as non‐responders [15], our cohort demonstrated a different pattern. All four pwCF in our series who carried canonical Class I alleles falling within the nonresponsive “true Class I” category in CFTR2/CFTR‐France experienced clinical benefit on long‐term ETI, including improvements in ppFEV1, BMI, and exacerbation frequency. This divergence suggests that some Middle Eastern Class I variants may not behave identically to their European counterparts and highlights the possibility of population‐specific, genotype‐modulator responsiveness not fully captured by current annotation frameworks. Our findings, therefore, add an important piece to the global picture: whereas the French data remain definitive for European Class I alleles, real‐world outcomes in underrepresented populations underscore the need for a more nuanced approach when determining ETI eligibility in pwCF with Class I genotypes.

Sweat chloride data were unavailable for most participants, a limitation reflecting regional laboratory constraints. Nevertheless, published registry and organoid studies suggest that sweat chloride reductions correlate well with clinical response and can serve as a pharmacodynamic biomarker for CFTR modulation.

In a prospective, multicenter trial involving 20 CF patients with the Class I minimal function N1303K mutation, while not directly comparable with our cohort, no significant reduction in sweat chloride was observed following ETI therapy, despite marked improvements in clinical outcomes [13]. This emphasizes the possibility that sweat chloride may not fully capture therapeutic responses in specific rare CFTR variants such as those seen in our cohort. This study′s retrospective design, small sample size, and single‐center setting limit its statistical power and generalizability, though the cohort provides valuable insights into the efficacy of ETI in these rare CFTR variants.

In conclusion, our results show that ETI significantly improves lung function, nutrition, and exacerbation rates, underscoring its benefits beyond F508del variants. These findings support expanding ETI access for rare variants based on functional response, particularly for underserved populations like those in the Middle East.

## Conflicts of Interest

The authors declare no conflicts of interest.

## Funding

No funding was received for this manuscript.

## Supporting information


**Supporting Information** Additional supporting information can be found online in the Supporting Information section. Table S1: The table provides individual‐level clinical characteristics and treatment responses for the 12 people with cystic fibrosis included in this study. The table details CFTR genotypes, functional class, prior modulator therapy, baseline and 12‐month ppFEV1 values, BMI changes, and annual pulmonary exacerbation frequency before and after initiation of elexacaftor/tezacaftor/ivacaftor (ETI). This dataset supports the descriptive analyses presented in the manuscript and allows for transparent comparison across genotypic subgroups.

## Data Availability

The data that support the findings of this study are available on request from the corresponding author. The data are not publicly available due to privacy or ethical restrictions.
