# Supplementary material for: Highly Effective Modulator Therapy in Cystic Fibrosis: Addressing Unusual Variants in the Middle East
Source: Pulm Med. 2025 Dec 26;2025:3622052. doi: 10.1155/pm/3622052 (PMC12767469; doi:10.1155/pm/3622052)
Supplement: Supplementary file 1 — Supporting Information Additional supporting information can be found online in the Supporting Information section. Table S1: The table provides individual‐level clinical characteristics and treatment responses for the 12 people with cystic fibrosis included in this study. The table details CFTR genotypes, functional class, prior modulator therapy, baseline and 12‐month ppFEV1 values, BMI changes, and annual pulmonary exacerbation frequency before and after initiation of elexacaftor/tezacaftor/ivacaftor (ETI). This dataset supports the descriptive analyses presented in the manuscript and allows for transparent comparison across genotypic subgroups. [file PM-2025-3622052-s001.docx]

Supplementary Table S1. Individual Clinical Data for 12 pwCF on ETI.

| Patient ID | Genotype | Class | Prior modulator | ppFEV₁ baseline (%) | ppFEV₁ 12 mo (%) | BMI baseline | BMI 12 mo | Exacerbations pre/post |
| --- | --- | --- | --- | --- | --- | --- | --- | --- |
| P1 | 1548delG/1548delG | I | IVA | 35 | 43 | 18.5 | 19.8 | 3 → 1 |
| P2 | 3120+1G>A/R1158X | I | IVA | 31 | 39 | 19.0 | 20.1 | 4 → 2 |
| P3 | 406-2A>G/406-2A>G | I | None | 29 | 38 | 17.9 | 19.4 | 5 → 2 |
| P4 | R1158X/1548delG | I | IVA | 32 | 39 | 20.3 | 21.2 | 3 → 1 |
| P5 | G27V/3849+10kbC>T | III/V | None | 30 | 42 | 18.7 | 20.2 | 4 → 1 |
| P6 | S549R/I1234V | III | IVA | 36 | 47 | 21.0 | 22.2 | 2 → 0 |
| P7 | S549R/S549R | III | IVA | 38 | 50 | 20.5 | 22.1 | 1 → 0 |
| P8 | I1234V/I1234V | III | IVA | 33 | 44 | 19.2 | 20.7 | 2 → 1 |
| P9 | I1234V/3849+10kbC>T | III/V | IVA | 35 | 46 | 21.4 | 22.9 | 2 → 0 |
| P10 | S549R/G27V | III | IVA | 37 | 45 | 19.6 | 20.8 | 1 → 0 |
| P11 | 3849+10kbC>T/406-2A>G | V/I | None | 27 | 36 | 18.0 | 19.1 | 5 → 3 |
| P12 | I1234V/R1158X | III/I | IVA | 34 | 42 | 20.1 | 21.3 | 2 → 1 |
